# Quint Rusman

**DOI:** 10.1111/nph.16160

**Published:** 2020-03-31

**Keywords:** flowering plants, flowers, herbivore‐induced plant responses, herbivore–pollinator interactions, plant reproduction, plasticity, Profile

Quint Rusman's poster ‘Settling on leaves or flowers: herbivore feeding site determines the outcome of indirect interactions between herbivores and pollinators’ won first prize at the 43^rd^ New Phytologist Symposium ‘Interaction networks and trait evolution’. See https://www.newphytologist.org/symposia/43 and the Meeting report by Frachon in this issue of *New Phytologist*, pp. 644–646.

## What inspired your interest in plant science?

I developed a keen interest in plant science during my BSc and MSc Biology at Wageningen University in the Netherlands. During my studies, I was introduced to the many interesting facets of plants, and was especially intrigued by their versatility. Plants are extremely plastic, and this is apparent in their physiology and ecology, in their interactions with both mutualistic and antagonistic species, and on a broader scale in the composition and dynamics of vegetation communities. I became astonished by the fact that plants can mediate interactions between different community members due to their plastic phenotype and this became the topic of my PhD studies entitled ‘Caught between friends and foes – plant‐mediated interactions between herbivores and flower visitors’. Plant responses that start at the molecular level to all kinds of biotic interactions can scale up to ecological consequences for numerous community members, being either mutualistic or antagonistic and are involved in very intimate or loose relationships with the roots, leaves and/or flowers of the plant. An important aspect that triggered, and still triggers, my love for plants was, and still is, working in the glasshouse with plants, and going out into the field to see and work with them in their natural settings. Seeing these interactions play out in nature is just fascinating.

## Why did you decide to pursue a career in research?

I think my personality guided me to a career in research, and especially to academia. I have a very broad interest, and love to explore various aspects of whatever topic interests me at the moment. I enjoy deciphering the biological world, from the very mechanistic perspective of the way in which organisms function, to the evolution and function of whole ecosystems. Science gives me an excellent platform to express these character traits. Science allows for a flexible way of working, providing freedom to reach the end goal of a certain project or question at your pace and via your way of working. Interestingly, the question or the end goal of a project might change during the process, and hence, you never know where you might end up, or what roads lie ahead. This is what I find fascinating about science.

Box 1

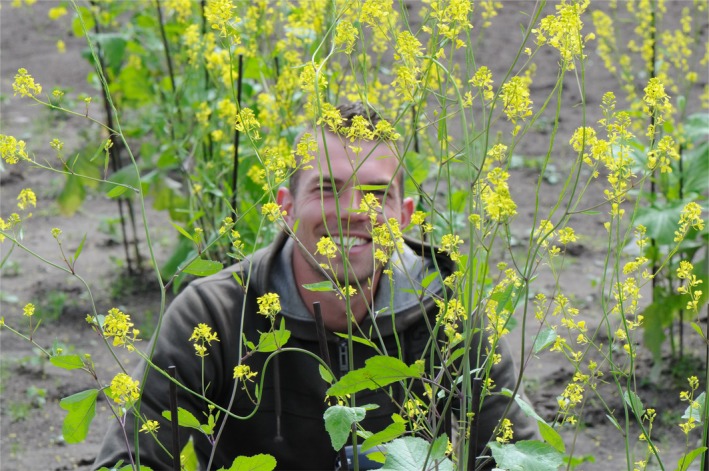

Quint Rusman was born on 9 July 1991 in Noordwijkerhout, the Netherlands. He moved to Wageningen to study Biology at Wageningen University. After finishing his Bachelor's degree in Biology with a focus on ecology, he started the Master programme in Biology at Wageningen University, with a focus on bio‐interactions, entomology and plant ecology. At the end of his MSc programme, Quint enrolled in the Experimental Plant Sciences Graduate Program, a program for excellent master students to write a PhD proposal and apply for funding. Together with Erik H. Poelman, Dani Lucas‐Barbosa, and Marcel Dicke of the Laboratory of Entomology at Wageningen University a proposal was written and it was successful: funding was granted. Starting in 2014 with his PhD project, Quint studied how attack by a range of herbivore species affects plant interactions with mutualistic and antagonistic flower visitors, the underlying herbivore‐induced changes in flower traits, and whether these interactions have plant fitness consequences. In a comparative approach using 10 different herbivore species, field, glasshouse, and laboratory work were combined. For the future, Quint aims to pursue a career in academia and continue studying how indirect interactions can drive ecological and evolutionary dynamics of complex communities, with special focus on plants and herbivore–flower‐visitor interactions. He is currently looking for a post‐doctorate position.

For more information on Quint, visit https://www.researchgate.net/profile/Quint_Rusman, or contact him at quint.rusman@hotmail.com.

ORCID: Quint Rusman https://orcid.org/0000-0003-0285-7967

Another important aspect for my career choice was the scientific atmosphere. I was exposed to the working environment of researchers in academic institutions during my two thesis projects for my Masters degree. I very much enjoy the work attitude of scientists, which includes lots of freedom, openness, passion about their research, and going the extra mile not because you have to, but because you want to. I enjoy sharing the knowledge I generate from my work with the people around me, both scientific and nonscientific, not just for the sake of knowledge, but so that the knowledge can be used to improve different aspects of society, and especially a sustainable living environment by including nature rather than exploiting it.

## What motivates you on a day‐to‐day basis?

From a young age I liked to discover new things. I particularly remember one time on a family camping trip near a lake where we brought a small rubber boat. Parts of the shore were covered with reeds through which some small waterways entered the lakes. I was very eager to discover something amazing within these reed‐filled waterways – maybe a cool island or a nest of turtles or water snakes. So we tried entering one of these waterways, despite my older brother urging us not to. Of course, our rubber boat got punctured by the reeds and we started sinking. This drive to uncover the unknown gets me out of bed every morning.

I also very much enjoy working with plants and insects, especially in spring and summer in the field, but also in autumn and winter in the glasshouse and laboratory. It is a treat to watch these guys go about their daily tasks, from little bees and funky looking syrphid flies that fight for the best flowers, to beautiful caterpillars and beetles enjoying the most juicy plant parts.

## Is there anyone that you consider to be a role model?

Many people have inspired me along the way so far. My PhD supervisor Erik H. Poelman is a great source of inspiration for me. Erik constantly encourages us to push the boundary of our current thinking, and always strives to perform novel and exciting experiments. While working on the frontier of science, I learned from him that it is essential to have a clear view of your aims and define the boundary before pushing it forwards. He inspires co‐operation and teamwork rather than competition. My other PhD supervisor Dani Lucas‐Barbosa showed me, among others, how strong one's passion for science can be and how to express this. She is an amazing writer who aims to transform each dataset in an exciting and novel way rather than a mere recital of outcomes. The supervisor of one of my Master thesis projects, David Kleijn, opened my eyes to the amazing world of pollinators. He inspired me not to see species as mere nodes in ecological networks, but as characters, with their personalities and backstories. Getting to know these characters might unveil the story you want to unravel before it even begins. Last, I want to name Joop Schaminée. Joop enlightened my world with vegetation science, and showed me that nothing can stop you from going out to explore nature, no matter what age you are or what difficulties you face. Joop is an incredible lecturer, with a fantastic ability to cover the boundaries between science and society, and teacher and student.

## What are your favourite *New Phytologist* papers of recent years, and why?

There are so many great *New Phytologist* papers that can be mentioned. One of the keystone papers in the development of my way of thinking about ecology and evolution is the review of Sharon Strauss and colleagues on the diffuse evolution of traits (Strauss *et al*., 2005). Although this paper was published quite some years back, it is still very relevant today. This paper manages to very clearly describe a rather abstract topic, and give sound instructions on how to measure such an abstract topic. I very much enjoy the work by Hans Jacquemyn and colleagues on terrestrial orchids and their associated mycorrhizal communities (Jacquemyn *et al*., 2014, 2015; McCormick & Jacquemyn, 2014). This work explores many new aspects of the interaction between orchids and mycorrhiza from a community perspective, and they use an elegant combination of fieldwork and statistical methods to expose these aspects. More related to my own field, I closely follow the work by Florian Schiestl and colleagues on floral volatiles and herbivore–pollinator interactions (Schiestl *et al*., 2014; Schiestl, 2015). Contrary to the community perspective of Hans Jacquemyn, Florian's approach to complex issues is to start simple and gradually increase complexity. This approach is both invigorating and rewarding, which is apparent in both his experimental (Schiestl *et al*., 2014) and theoretical work (Schiestl, 2015). Some more recent papers I wish to highlight are the works by Øystein H. Opedal on the evolutionary potential of plant traits (Opedal, 2019), and by Lucille Chretién and colleagues on plant‐mediated interactions with flowering plants (Chrétien *et al*., 2018). Both papers highlight the fact that flowers are unique plant organs, and although plants function as one integrated unit, plant responses to herbivory are different in flowers compared to leaves (Chrétien *et al*., 2018), and flowers have different evolutionary potential compared to vegetative tissues (Opedal, 2019).

## What is your favourite plant, and why?

I do not have one favourite plant, and how can you when you have to choose from the incredible diversity of plant forms and colours. I rather enjoy vegetation communities, and especially dune communities and wet tropical forests (quite the contrast I know, another aspect to wonder about!). As a kid I grew up in a small village near the west coast of the Netherlands, and my parents would often take us strolling through the dunes. I have grown to appreciate the dune vegetation, maybe partly because of the nostalgia, but also because of the many adorable plants such as *Viola curtisii* and *Liparis loeselii*. Wet tropical forests sparked my imagination as a child, and they still do to this day. The sheer diversity that can be found in tropical forests amazes me, and during every hike I feel there are new plants and animals to be found behind the next tree along the path. And not just any plant or animal, but out‐of‐this‐world ones like giant corpse flowers (*Rafflesia* spp.), funky ferns and mosses with poison frogs (Dendrobatidae) and geckos (*Phelsuma* and *Lygodactylus*) hiding in between, and beautiful orchids.

Not surprisingly from the earlier section, I have a soft spot for orchids, especially terrestrial ones. I am astonished by the ecology and evolution of orchids, and amazed by their diversity with around 26 000 species. Terrestrial orchids have a fascinating life cycle. It starts as a tiny dust seed that germinates in a protocorm (underground seedling) that can spend multiple years underground. During this early phase, the orchid engages in one or multiple interactions with mycorrhizal fungi and, in contrast to most mycorrhizal–plant interactions, the orchid may actually be a parasite in some cases! The short time that most terrestrial orchids are aboveground is spent on giving exquisite flower shows and seducing either a single species of insect, or more than a hundred, to visit their flowers by providing sweet rewards, or merely the illusion of food or sex.
